# Special issue on “calcium signaling”

**DOI:** 10.52601/bpr.2024.240061

**Published:** 2024-12-31

**Authors:** Zong Jie Cui

**Affiliations:** 1 College of Life Sciences, Beijing Normal University, Beijing 100875, China

To commemorate the occasion of the establishment of The Section for Calcium Signaling in The Chinese Biophysics Society on 21 July 2023 in Changsha, Hunan Province, the editorial office of *Biophysics Reports* decided to call for a Special Issue on Calcium Signaling, the Special Issue was now published in October 2024. As stressed in the Preface to that Special Issue (Wang 2024), the divalent cation Ca^2+^ plays a plethora of physiological roles from birth to death of all living organisms.

Research on calcium signaling has a relatively late start in China. Most of the works started in the early 1990s, and the number of publications reached double digits only in 1993 (Fig. 1). According to the analysis of the Web of Science database, works on calcium signaling started in China in 1982, two years after a major review on the role of calmodulin was published in the journal of *Science* (Cheung 1980). Early works had been carried out on varied aspects of calcium signaling, in different universities and research institutes. In the following, a few examples are highlighted.

**Figure 1:**
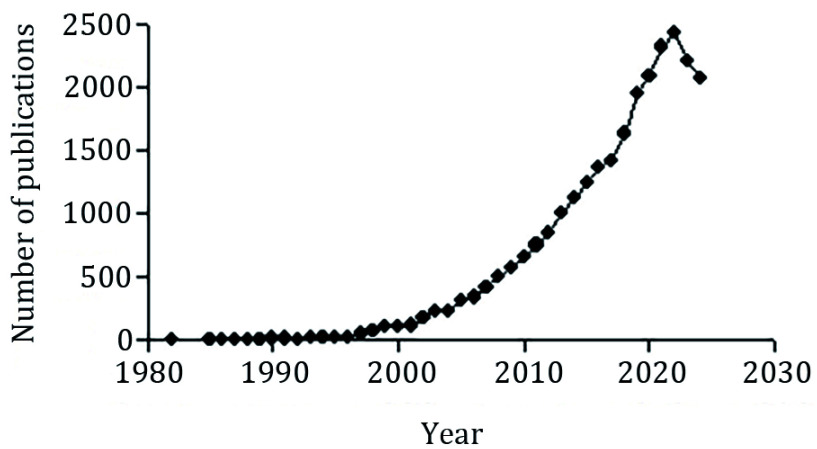
Number of publications on calcium signaling by authors from China. Data are obtained from Web of Science, on 11 Nov., 2024

The extracellular plant cell wall calmodulin was extensively studied at Hebei Normal University (Ye *et al*. 1989). The calcium-calmodulin-CaMKII signaling pathway was carefully studied at Xuzhou Medical University (Tang *et al.* 1991). A series of extensive works on calcium-dependent phosphatase calcineurin lasting three decades was initiated at Beijing Normal University (Meng and Wei 1994). Calcium signaling in the skeletal muscle cells was carried out at the Shanghai Institute of Physiology (Fu and Zhu 1993; Zhu *et al*. 1986). Multiple works on calcium release were published by the Xue group at Beijing Normal University (Xue *et al*. 1993, 1994; Cui *et al.* 1995), as reviewed in this Special Issue (Cui 2024). The important role of transmembrane calcium gradient was investigated at the Institute of Biophysics (Huang *et al*. 1994). Calcium signaling in reproduction was done at the Institute of Development (Deng *et al*. 1997), and Beijing Institute of Materia Medica (Qiao *et al*. 1994). Investigators were working on calcium signaling in Hong Kong SAR (Webb *et al*. 1997), and in Taiwan province (Chueh and Kao 1994; Yang, *et al*. 1994). Other than intrinsic or cellular physiology of calcium signaling, towards the late 1990s, it had been found in a bilateral work that photodynamic therapy, a novel mode of treatment modality for cancer, at lowered doses, could trigger the physiological process of calcium oscillations (Cui and Kanno 1997), laying the foundation for the eventual discovery of G protein-coupled receptor activated by singlet oxygen (GPCR-ABSO) (Jiang *et al*. 2018).

Several special group meetings on calcium signaling are held regularly over the globe. Like the overseas calcium meetings, The Chinese Symposium on Calcium Signaling (CSCS) has been held regularly since 1987. The first five such meetings (held at the cities of Xuzhou, Tianjin, Shijiazhuang, Nanjing, and Xuzhou) on Calcium and Cellular Function were organized every three years under the auspices of the Chinese Society of Cell Biology, anchored by the late Professor Shao Bai Xue (Cui 2024). Starting from the Qingdao meeting the symposium has been reorganized to be held every two years under the auspices of the Chinese Biophysics Society (Qingdao 2006, Chair: Zhuan Zhou; Yichang 2008, Chair: Zong Jie Cui). Subsequent meetings are held regularly every two years (Beijing 2010, Co-Chairs: Heping Cheng, Zhuan Zhou; Huangshan 2012, Chair: Feng Gao; Yichun 2014, Co-Chairs: Xu Hui Zeng, Jie Zheng; Zunyi 2016, Co-Chairs: Bi Guang Tuo, Jenny Yang; Shenyang 2018, Co-Chairs: Liying Hao, Lu Yang Wang), but interrupted by the COVID’19 pandemic, for the 13^th^ meeting to be delayed by four years, to be held in 2024 in Wuhan (Co-Chairs: Jian Feng Liu, Hao Xin Xu). Zhuan Zhou, Mike Xi Zhu, and the succeeding chairs played an essential role for this meeting to continue. These calcium meetings facilitated cooperation, to eventually cultivate into the official establishment of the Section for Calcium Signaling in the Chinese Biophysics Society, and the publication of the Special Issue on Calcium Signaling (Wang 2024).

Figure 1 shows that the number of works published on calcium signaling from China increased to three digits in 1999, to four digits in 2014, with the number peaking at 2430 in 2022. The most recent 20 works published so far involved important topics such as glucocorticoid receptors, calcium-binding protein S100A16, CaMKII, ER stress-ATF6-Ca^2+^ axis, TRPM7, ferroptosis, and NF-*k*B.

## Conflict of interest

Zong Jie Cui declare that they have no conflict of interest.
